# Development and trainability of agility in youth: A systematic scoping review

**DOI:** 10.3389/fspor.2022.952779

**Published:** 2022-09-08

**Authors:** Lutz Thieschäfer, Dirk Büsch

**Affiliations:** Institute of Sport Science, Carl von Ossietzky University Oldenburg, Oldenburg, Germany

**Keywords:** reactive agility, unplanned change-of-direction, multi-directional speed, pediatric performance, youth fitness, maturation

## Abstract

**Background:**

Agility is acknowledged as a crucial factor of performance in various open skill sports in both adult and youth athletes. However, despite its significance for sports performance the development and the trainability of agility are under-researched within the pediatric literature. A systematic scoping review was considered most appropriate to provide researchers and practitioners with an overview of the current body of literature approaching agility in youth.

**Objectives:**

The objectives of this scoping review were to map the extent, range, and nature of existing evidence regarding trainability and “natural” development of agility and to summarize corresponding study results.

**Methods:**

The scoping review protocol was pre-registered at Open Science Framework. Systematic searches were conducted using the databases PubMed, Scopus, ProQuest, Web of Science, SURF, and SPONET to identify sources covering agility in youth. Among other inclusion criteria, only references applying unplanned agility concepts were included.

**Results:**

Ultimately, 41 reports were included comprising 23 observational studies, 14 experimental studies, and 4 references of secondary research. A total of 3,087 subjects were assessed in the included studies. Subject groups were predominantly male, above 10 years of age, and soccer athletes. Outcomes of observational studies indicate an effect of age and maturation on agility performance resulting in a non-linear “natural” development of agility. Furthermore, relationships between contributing perceptual-cognitive factors and agility performance tend to increase with progressing age, whereas relationships between physical factors and agility performance diminish. Evidence of training studies suggests that agility is trainable in youth, albeit with various underlying mechanisms.

**Conclusions:**

This systematic scoping review is the first mapping of the body of literature about agility in youth. It outlines the current evidence base, reveals research gaps, and points out future directions to support researchers and practitioners in this field. Although, increasing research activity in this field is discernible, agility research in youth is still in its infancy. Considering the significance of agility for sports performance, future research is postulated to design evidence-based strategies for long-term agility development in young athletes.

## Introduction

The ability to execute changes in movement patterns and directions as rapidly as possible is a desired quality highly regarded by coaches and athletes across a wide array of sports, such as field, court, and combat sports (1–3). This quality is considered agility if the executed movement is an open skill task that is performed in response to a stimulus (e.g., opponent, teammate, or ball) (4). Equally, in young athletes, well-developed agility is acknowledged as a critical factor for success as it contributes to athletes' performance in various open skill sports (5). Accordingly, the development of agility competencies in youth is deemed necessary by strength and conditioning coaches (6). Moreover, agility attained recognition by its implementation in long-term athletic development models, which guide a systematic enhancement of skills and abilities throughout childhood and adolescence (7, 8).

Despite the generally accepted high significance of agility for sports performance, Lloyd and Oliver (7) stated that “agility is arguably one of the most under-researched fitness components within the pediatric literature […]”. Due to a dearth of research, unraveling the impacts of age and maturation on agility performance is difficult, and assertions about agility's optimal trainability are generally speculative (5). This situation is further exacerbated by two facts: First, unlike the paucity of agility research, a plethora of research is devoted to change-of-direction (COD) ability, which was formerly described as the integrated movement component of agility performance (9). In contrast to agility tasks, COD movements are without the requirement to react to a stimulus; thus, they are pre-planned. Furthermore, recent research indicates relatively low statistical commonalities of agility and COD performances, which are considered distinct qualities (10–17). Consequently, findings of COD research cannot automatically be extrapolated to agility (14). Second, most agility research was conducted in an adult population. The defining difference between youth and adult athletes is the consequences of growth and maturation, which are paramount in pediatric exercise science (18). Hence, equally like the limited transferability of COD research to agility, research findings of agility training ascertained in the adult population cannot implicitly be applied to young athletes (19).

Growth and maturation follow individual (in timing and tempo) and non-linear trajectories with alternating periods of stagnant changes and abundant fluctuations (20, 21). These volatile stages of growth and maturation are likely to affect the trainability of youth athletes (22), which refers to the responsiveness to a specific instructional, practice, and/or training stimulus (23). Thus, mechanisms and effects of specific and non-specific agility training probably vary with biological age. Recommendations regarding age-appropriate agility training are mainly speculative due to the sparse literature in this field (5). Nonetheless, the trainability of agility can at least be approximated by examining the trainability of the main factors that determine agility performance. Several deterministic models of agility performance have been proposed (4, 9, 13, 24–27), which have close resemblance regarding their organismic components (i.e., perceptual, cognitive, physical, and motor control factors). The contribution of these factors to agility performance is variable and presumably also growth and maturation dependent (13). Given the proven trainability of strength (28, 29) and speed (30), as well as motor (23) and anticipation skills (31) in youth and provided that these enhancements potentially transfer to agility, it might be assumed that agility is likewise trainable (5), even though the extent and underlying mechanisms differ at different developmental periods.

Cross-sectional data suggests that agility performance enhances “naturally” (i.e., apart from training) with increasing age, but with significant increases from childhood to early adolescence and a near-plateau in mid-adolescence (32). Therefore, training adaptations (in agility performance) are not solely attributable to the impacts of exposed training stimuli but also to the natural developmental processes of the young athlete (33). Thus, the origins of gains in agility performance are indistinct. It is difficult to differentiate between “natural” development and exercise-induced adaptations; this complicates the understanding of trainability in youth athletes (19, 34). In conclusion, it is imperative to consider growth and maturation aspects in youth athlete development (e.g., by regular monitoring of stature and body mass) (33), as they affect both the trainability and the “natural” development of agility throughout childhood and adolescence.

A decade has passed since the above-mentioned statement of Lloyd and Oliver (7), and it is about time to reevaluate the state of agility research in the pediatric exercise science literature. The conduct of a systematic scoping review was considered the most appropriate method to address the research question: What is known from the academic literature about the “natural” development and trainability of agility in youth? Agility has been subject to several systematic reviews approaching contributing factors (35, 36), effects of training interventions (36), and testing procedures (3, 36–38). However, the effects of growth and maturation were not explicitly considered in these reviews. Although trainability and “natural” development of agility in consideration of growth and maturation have been narratively reviewed (5, 30, 39), a systematic approach is yet to be carried out.

The scoping review had the following objectives: (1) to examine the extent, range, and nature of literature approaching agility in the youth population; (2) to reveal gaps in the literature to guide future research endeavors; and (3) to map and outline evidence regarding trainability, “natural” development, and contribution of underlying key factors of agility performance in consideration of maturation.

## Methods

A systematic scoping review was conducted in accordance with the Preferred Reporting Items for Systematic Reviews and Meta-Analyses extension for scoping reviews (PRISMA-ScR) (40) guidelines to address the research questions. This study was not approved by an institutional review board as the authors collected and synthesized data from previous studies in which informed consent was already obtained. Thus, this study is exempt from ethical approval. The study protocol was drafted using an adapted registration form for the PROSPERO database and revised by the authors. The final protocol (41) was pre-registered with the Open Science Framework^1^ on Jul 21, 2021.

### Search strategy

Six bibliographic databases (ProQuest, PubMed, Scopus, Web of Science, SPONET, SURF) were selected as appropriate sources for the systematic literature search, which was completely performed on Jul 22, 2021. The title, abstract, and keyword fields were searched with search terms that included synonyms for “agility” (agility OR unplanned move^*^ OR unplanned task^*^ OR unplanned sidestep^*^ OR reactive move^*^ OR reactive task^*^ OR reactive cut^*^) AND “youth” (child^*^ OR adolescen^*^ OR youth^*^ OR puber^*^ OR kid^*^ OR teen^*^ OR girl^*^ OR boy^*^ OR young^*^ OR junior^*^). In addition, the search string utilized in Germanophone databases (SPONET and SURF) was extended with respective German search terms (agilität AND (kind^*^ OR jugend^*^ OR nachwuchs^*^ OR mädchen OR jung^*^)).

### Eligibility criteria

Eligible publications had to conform to several inclusion criteria: (1) Exclusively articles from peer-reviewed journals, (edited) books, conference works, and dissertation theses, and (2) written in English or German language were considered. It was expected that the restriction of the publication type would ensure a minimum quality of the included sources, (3) The applied concept of agility had to align with the definition of (4) (4): “a rapid whole-body movement with change of velocity or direction in response to a stimulus.” Thus, reports applying pre-planned agility concepts (i.e., COD speed [CODS]) were excluded. In addition, agility concepts involved ground-based movements; aquatic or on-ice agility was out of scope. (4) Outcome(s) had to be relevant to the review question (i.e., outcome(s) had to be related to “natural” development or trainability of agility in youth). For example, (42) evaluated a new agility test in young tennis players and assessed agility performance in youth athletes. However, this publication does not provide information about the development or trainability of agility and is consequently deemed ineligible. (5) Effects of maturation are prominent from birth until early adulthood. Therefore, findings in early childhood were out of the scope of the present review, and effects of maturation after adolescence were expected to be neglectable. Therefore, the population addressed in the included studies had to be between 6 and 19 years of age. (6) The presence of diseases, injuries, or certain disabilities might substantially impact reported results. Thus, only sources assessing healthy subjects were considered.

### Selection strategy

Duplicates were removed after the search process using EndNote 20 (Clarivate, Philadelphia, USA). Afterward, records and reports were screened by a two-step approach (title and abstract screening, full-text screening) by one investigator using DistillerSR (Evidence Partners, Ottawa, Canada). It was not necessary to review each publication by more than one expert due to the objectivity of the inclusion criteria. Uncertainties in the selection process were resolved through consultation and discussion with a second researcher. Title and abstract screening were merged in a single step because it was anticipated that in most cases, a clear identification of the applied agility concept will be impossible by screening the publication title alone. If title and abstract provided insufficient information for a decision, full text was retrieved and further reviewed for eligibility verification. Reference lists of included reports were manually searched for additional potentially eligible publications, which might have escaped the search strategy. Data sets of included publications for each step are accessible at Open Science Framework^2^ (43) for methodological transparency and reproducibility purposes.

### Data extraction and synthesis

Data were extracted by a single reviewer, discussed with a second researcher and categorized in observational, experimental, and secondary research. Publication characteristics (e.g., type, year, language), subject characteristics (e.g., age, maturation stage, sex, type of sport, performance level), study characteristics (e.g., sample size, measurements), and main outcome(s) were extracted and charted using Microsoft Excel (Microsoft Corp, Redmond, USA) spreadsheets. The stimuli used in the agility tests were categorized based on their specificity into generic (e.g., flashing lights, arrows, or movement instructions from a screen), video (i.e., footage of human movements), and human (i.e., live tester that initiates the movement to which the athlete must respond) stimuli. A critical appraisal or risk of bias assessment was not performed, following the recommendations of Peters et al. (44). The findings of included sources were summarized in a narrative synthesis.

Cohen's recommended criteria were used to characterize effect sizes (45). If effect sizes were not provided, Cohen's *d* was calculated *post-hoc* according to the recommendations of Cumming (46) for dependent samples. Missing confidence intervals of effect sizes were calculated in accordance with Hedgesand Olkin (47).

## Results

**Figure 1:**
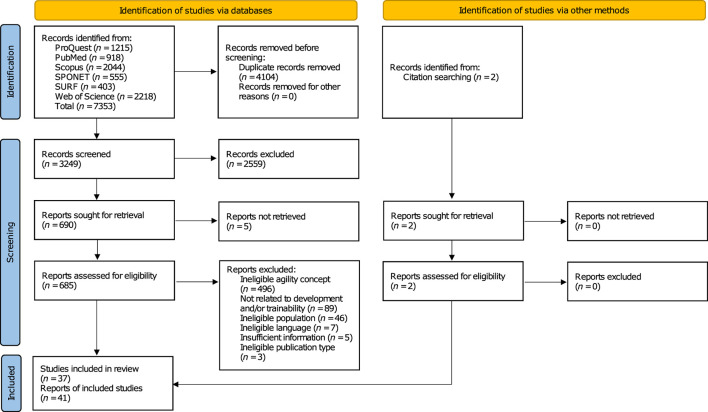
PRISMA 2020 flow diagram of the search and selection process (48).

The search and selection process results are illustrated in detail in a flow diagram (Figure 1). A total of 7,353 records were identified from the bibliographic database search. Titles and abstracts of 3,249 publications were screened after eliminating duplicate records. The eligibility of 685 remaining reports was assessed, which resulted in the inclusion of 39 reports. The exclusion reasons were distributed as follows: ineligibility regarding agility concept (*n* = 496), population assessed (*n* = 46), publication language (*n* = 7), and publication type (*n* = 3), as well as missing content-related affiliation (*n* = 89) and insufficiency of provided information (*n* = 5). Searches of reference lists of the included publications revealed two additional eligible reports, whereby 41 reports were ultimately included in the current scoping review.

### Publications' characteristics

**Figure 2:**
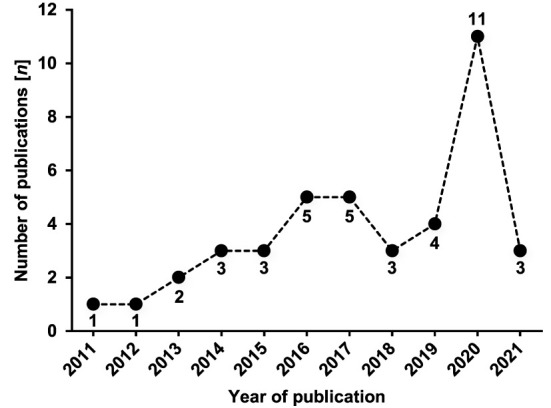
Timeline of publications 2011–2021.

The included publications (*n* = 41) were composed of several journal articles (*n* = 34) (32, 39, 49–80), conference abstracts (*n* = 3) (81–83), contributions in edited volumes (*n* = 2) (5, 30), one PhD thesis (*n* = 1) (84), and one monograph (*n* = 1) (85). Figure 2 illustrates the growth of publications per year, with publication years ranging from 2011 until 2021. The publications are categorized in reports of observational studies (*n* = 23) (51, 52, 59, 61, 64–69, 71–73, 75–78, 80–85), reports of experimental studies (*n* = 14) (49, 50, 53–58, 60, 62, 63, 70, 74, 79), and secondary research (*n* = 4) (5, 30, 39, 85). Relevant data extracted from respective underlying studies (*n* = 37) are presented for observational studies in Table 1 and for experimental studies in Table 2. Research was conducted in 17 different countries. The vast majority of studies (*n* = 30) (32, 51, 52, 55–57, 59–62, 64–73, 75–84) originated in Europe, with most contributions coming from Slovakia (*n* = 8) (32, 59, 64, 66, 67, 69, 71, 80). Other countries of origin were Australia (*n* = 3) (49, 54, 63), Taiwan (*n* = 1) (74), Tunisia (*n* = 2) (53, 58), and USA (*n* = 1) (50).

**Table 1:** Characteristics and outcomes of included observational studies.

**Study details**	**Subjects**				**Methodological details**		**Outcomes**
**Study Publication Type**	**Sport Level**	**Sex**	**Group** ***n***	**Age (years)**	**Age from PHV (years)**	**Agility test Stimuli AT**^a^ **and RT**^b^ **separated**	**Main findings**
(78) Journal article	Soccer highest national competition	m	U15 = 27 U17 = 25 U19 = 23	14.7 ± 0.6 16.2 ± 0.7 18.8 ± 0.7	Not assessed	Y-shaped Generic, human Yes	Age group effects were observed for AT and RT in tests with human stimuli [*F*_(1, 144)_ = 8.99, *p* ≤ 0.01, η^2^ = 0.20; *F*_(1, 144)_ = 16.27, *p* ≤ 0.01, η^2^ = 0.31, respectively]. AT and RT were significantly lower in U19 (*p* ≤ 0.05) compared to younger age groups. Tests with light stimuli failed to discriminate between age groups.
(71) Journal article	Soccer ?	?	U12 = 15 U14 = 14 U16 = 12	11 13 15	−2.2 ± 0.5 −0.1 ± 0.7 1.9 ± 0.4	Y-shaped Generic No	ANOVA and ANCOVA (PHV as covariate) showed significant main effects in AT (*F =* 49.6, *p < * 0.01; *F =* 6.5, *p < * 0.01, respectively). Agility performance was significantly better in U14 compared to U12, and U16 outperformed U12 and U14 as well (*p < * 0.05, *g =* 0.87; *p < * 0.01, *g =* 3.99; *p < * 0.01, *g* = 2.57, respectively). FMS total score and AT were not significantly correlated.
(65) Journal article	Soccer ?	m	U10 = 125 U12 = 125 U14 = 125 U16 = 125	?	Not assessed	Dribbling agility test Generic No	Main effects for group were observed in dribbling AT [*F*_(3, 493) =_ 88, *p* <0.01, η^2^ = 0.32]. *Post-hoc* tests revealed differences between all groups (*p* <0.01).
(82) Conference abstract	Soccer Top division	?	U14 = 17 U15 = 16 U16 = 23 U17 = 16	14.3 ± 1.1 for entire sample	Not assessed	Reactive multidirectional speed test Generic No	Significant effects of age on AT were observed (*p* <0.001). *Post-hoc* test revealed that U15, U16 and U17 players outperformed U14 players in AT. No differences in performance were observed between U15, U16, and U17 players.
(72) Journal article	Soccer Elite	m	U12 = 8 U13 = 11 U14 = 15 U15 = 6 U16 = 10 U18 = 13	11.9 ± 0.3 12.7 ± 0.4 13.9 ± 0.2 14.9 ± 0.1 15.8 ± 0.2 16.7 ± 0.5	−1.9 ± 0.2 −1.0 ± 0.8 0.1 ± 0.5 1.0 ± 0.8 2.2 ± 0.4 ?	Reactive repeated-sprint test Generic No	AT progressively decreased from U12 to U16 (*p* <0.01, *d =* 1.0–1.9). No significant differences were found between U16 and U18). No between group differences were evident
**Study Publication Type**	**Sport Level**	**Sex**	**Group** ***n***	**Age (years)**	**Age from PHV (years)**	**Agility test Stimuli AT**^a^ **and RT**^b^ **separated**	**Main findings**
							after inclusion of age at PHV as covariate (*p* > 0.05, *d* <0.3). AT and Arrowhead agility time correlated significantly in U12 and U13 (*r* = 0.85, *p* ≤ 0.05; *r* = 0.73, *p* ≤ 0.05, respectively)
(84) PhD thesis	Soccer Top level	?	U12 = 31 U14 = 23 U17 = 32	13.6 ± 2.0 for entire sample	?	Y-shaped Generic No	U14 and U17 significantly outperformed U12 in AT (*p* <0.01). Various significant relationships of moderate to large magnitudes (*r* = |0.43-0.77 |) were observed between measures of jumping, CODS, sprint, and AT across the age groups.
(61) Journal article	Soccer ?	?	U12 = 39 U14 = 42 U16 = 70 U18 = 35	11.5 ± 0.5 13.4 ± 0.4 15.6 ± 0.4 17.5 ± 0.3	Not assessed	Y-shaped Generic Yes (REAC-INDEX)	Significant differences between age groups were found in AT and REAC-INDEX [*F*_(3, 182)_ = 5.59, *p* <0.05, η^2^ = 0.08; *F*_(3, 182)_ = 11.40, *p* <0.01, η^2^ = 0.16, respectively]. U12 was outperformed by older age groups regarding AT (*p* <0.01). U14 AT was inferior to U18 AT (*p* <0.05). No differences in AT were observed between U16 and U18 players. REAC-INDEX was significant different in all *post-hoc* group comparisons (*p* <0.01). Various significant correlations between CODS tests, REAC-INDEX and AT across the age groups were reported (*r* = |0.40-0.98|).
(81) Conference abstract	Soccer Highly trained	m	U15 = 25 U17 = 27 U19 = 23	14.4 ± 0.6 16.2 ± 0.7 18.3 ± 0.7	Not assessed	Y-shaped Generic, human No	No significant differences in AT between the age groups were evident.
(80) Journal article	Soccer ?	m	U11 = 20 U12 = 19 U13 = 18 U14 = 18 U15 = 19 U16 = 18	11.2 12.3 13.4 14.3 15.1 16.4	Not assessed	FITRO Agility check Generic No	Descriptive statistics were presented. The level of AT is almost identical in U11 and U12. A rapid improvement in agility performance was recorded in U13 with subsequent slighter increases in
**Study Publication Type**	**Sport Level**	**Sex**	**Group** ***n***	**Age (years)**	**Age from PHV (years)**	**Agility test Stimuli AT**^a^ **and RT**^b^ **separated**	**Main findings**
							U14 to U16. Trivial correlations were observed between CODS and AT in U11 and U12 players (*p* <0.05).
(66) Journal article	Soccer ?	?	U12 = 13 U13 = 12 U14 = 15 U15 = 17	?	Not assessed	FITRO Agility check Generic No	Descriptive statistics were presented. AT are almost stable between U12 and U13. Between U13 and U14 agility performance increased rapidly and plateaued between U14 and U15. Significant correlations between AT and CODS were observed in U12 (*ρ =* 0.79, *p* <0.01).
(67) Journal article	? ?	mx	Girls = 41 Boys = 59	17.5 ± 1.2 for entire sample	Not assessed	Y-shaped Generic No	Boys performed significantly better than girls in the agility test (*U* = 778.5, *z* = −3.02, *p* <0.01, *d* = 0.27). No statistically significant relationship was observed between agility performance and cognitive abilities assessed by a stroop test.
(59) Journal article	Table tennis	f	Mini cadet = 9 Cadet = 6 Junior = 8	13.2 *Mdn* 14.6 *Mdn* 16.6 *Mdn*	Not assessed	Modified FITRO Agility check Generic No	Group comparisons indicate no statistically significant differences of AT between groups (*p* > 0.05).
(69) Journal article	None N/A	m	U12 = 20 U13 = 27 U14 = 26 U15 = 27	11.0 ± 0.3 12.0 ± 0.3 13.0 ± 0.3 14.0 ± 0.3	Not assessed	FITRO Agility check Generic No	AT differed significantly between age groups [*F*_(3, 96)_ = 17.29, *p* <0.001]. U12 was outperformed by all older groups (*p* <0.01) and U13 was outperformed by U15 in AT (*p* <0.05).
(51) Journal article	Soccer Selected talented players, first division	?	U14 = 13 U19 = 21	14.1 ± 0.3 18.0 ± 0.9	Not assessed	Goalkeeper reaction and action velocity test Generic No	Various agility performance measures were significantly better in U19 goalkeepers (*p* <0.01; *d* = 1.57-2.16). Some measures of agility performance were significantly correlated with CMJ performance in U14 goalkeepers (*r* = −0.63 to −0.77).
**Study Publication Type**	**Sport Level**	**Sex**	**Group** ***n***	**Age (years)**	**Age from PHV (years)**	**Agility test Stimuli AT**^a^ **and RT**^b^ **separated**	**Main findings**
(73) Journal article	Soccer ?	m	U13 = 29 U15 = 30	13.4 ± 1.3 for entire sample	?	Y-shape with kick and return Generic No	Agility performance was significantly higher in the older group (*t* = 3.96, *p* <0.001, large ES differences). Performance measures, i.e., 20 m sprint, CODS, and CMJ were correlated with agility performance in U15 only (*r* = |0.49-0.64|, *p* <0.01). Multiple regression analysis for AT was significant in U15 only (*p* = 0.01), with predictors of sprint, jump, and CODS explaining 33% of variance.
(52) Journal article	Soccer ?	m	U11 = 10 U13 = 9 U16 = 11	11.2 ± 0.5 13.2 ± 0.2 15.6 ± 0.7	—2.78 ± 0.4 −1.44 ± 0.8 1.25 ± 0.4	Y-shape Generic No	U16 players significantly outperformed younger U11 and U13 players in agility performance (*p* <0.05, *d* > 1.2; *p* <0.05, *d* = 1.82, respectively). The U13 players were significantly more mature than U11 players, but they did not perform significantly better in agility. No significant differences in AT between the age groups were evident when maturation was considered as a covariate (*p* > 0.05). Agility performance and maturation were significantly related (*r* = 0.58, *p* <0.01). Agility was significantly related in 4 of 7 fundamental movement skills measures (*r* = −0.40 to −0.60). In-line lunge performance was identified as the primary predictor of agility performance in a stepwise multiple regression analysis (adjusted *R*^2^ = 38%).
(68) Journal article	Soccer Highest competition level	m	U17 = 10 U19 = 10	16.5 ± 0.7 17.5 ± 1.0	Not assessed	Soccer specific reactive agility test Generic No	AT of 3 different agility test protocols were significantly correlated with CODS (*r* = 0.50–0.63, *p* <0.05). Both age groups significantly differed in
**Study Publication Type**	**Sport Level**	**Sex**	**Group** ***n***	**Age (years)**	**Age from PHV (years)**	**Agility test Stimuli AT**^a^ **and RT**^b^ **separated**	**Main findings**
							CODS and in 2 of 3 agility performance tests (*t* = 2.14, *p* <0.05, ES = 0.96, 95% CI [0.48, 1.43]; *t* = 2.42, *p* <0.05, ES = 1.1, 95% CI [0.54, 1.62]) but not in any other investigated conditioning capacity.
(75) Journal article	Basketball, handball, volleyball Talented players	mx	Girls = 157 Boys = 149	13–15	Not assessed	Five-time shuttle run to gates test Generic No	Multiple linear regression analysis was conducted (adjusted *R*^2^ = 0.24, *F* = 10.82, *p* <0.001) revealing 3 independent variables (i.e., gender, age, and training experience) which were significantly associated with agility performance (β = −0.46, *p* <0.001, 95% CI [−0.58, −0.34]; β = −0.30, *p* <0.001, 95% CI [−0.42, −0.18]; β = −0.11, *p* <0.05, 95% CI [−0.21, −0.01], respectively). Further regression analyses for girls and boys were carried out (adjusted *R*^2^ = 0.14, *F* = 3.43, *p* <0.001; adjusted *R*^2^ = 0.12, *F* = 2.94, *p* = 0.01, respectively). Age, training per week, training experience and body mass significantly affected agility performance in girls (β = −0.18, *p* <0.05, 95% CI [−0.37, 0.00]; β = −0.18, *p* <0.05, 95% CI [−0.35, 0.00]; β = −0.18, *p* <0.05, 95% CI [−0.34, −0.03]; β = 0.28, *p* <0.05, 95% CI [0.04, 0.51], respectively). Age and correct reactions in a peripheral perception test explained some variability in agility performance in boys (β = −0.50, *p* <0.001, 95% CI [−0.73, −0.27]; β = −0.25, *p* <0.05, 95% CI [−0.46, −0.04], respectively).
**Study Publication Type**	**Sport Level**	**Sex**	**Group** ***n***	**Age (years)**	**Age from PHV (years)**	**Agility test Stimuli AT**^a^ **and RT**^b^ **separated**	**Main findings**
(83) Conference abstract	Soccer ?	m	U11 = 14 U15 = 12 U19 = 12	?	Not assessed	Soccer-specific reactive agility test with and without ball dribbling Video, generic No	Performance in the agility tests were significantly lower in U11 compared to the older groups (*p* <0.05).
(76) Journal article	Futsal Professional	m	Juniors = 18 Seniors = 26	18.9 ± 1.1 28.1 ± 5.2	Not assessed	Y-shape with kick and return Generic No	Agility performance was not significantly different between junior and senior futsal players (*p* > 0.05).
(64) Journal article	Basketball, handball, soccer, volleyball	m	U15 = 60 U17 = 57	15.0 ± 1.9 for entire sample	Not assessed	FITRO Agility check Generic No	Descriptive statistics and correlations were presented. Means of the agility test were higher in the younger age group.
(77) Journal article	Soccer Competitive level	m	41 in total	14.4 ± 0.5	0.74± 0.3	Y-shape with kick and return Generic No	Significant correlations were identified between agility performance and measures of sprint, jump and CODS (*r* = |0.42–0.55|, *p* <0.01), as well as age and maturity offset (*r* = −0.35, *p* <0.05; *r* = −0.36, *p* <0.05, respectively). Multiple regression calculation with maturity offset, body mass, and conditioning capacities as predictors did not reach statistical significance (*p* > 0.05).
(32) Journal article	? N/A	mx	553 in total *n* = 44-48 per age group	7-18	Not assessed	FITRO Agility check Generic No	AT decreased with increasing age. This decrease was rather steep from ages 7 to 10 and from ages 10 to 14 followed by a leveling off from 14 to 18 years of age.

ANOVA, analysis of variance; ANCOVA, analysis of covariance, AT, agility time; CMJ, counter movement jump; CODS, change-of-direction speed; ES, effect size, f, female; FMS, Functional Movement Screen; m, male; mx, mixed group of both sexes; N/A, not applicable; PHV, peak height velocity; RT, response time; ?, information not provided.

a Time to fully complete the agility test.

b Timeframe from the onset of the stimulus until the initiation of the response movement.

**Table 2:** Characteristics and outcomes of included experimental studies.

**Study details**	**Subjects**					**Methodological details**		**Outcomes**
**Study Publication Type**	**Sport level**	**Sex**	**Group** ***n***	**Age (years)**	**Age from PHV (years)**	**Agility test Stimuli AT**^a^ **and RT**^b^ **separated**	**Treatment**	**Main findings**
(79) Journal article	Athletics, basketball, handball, soccer, table tennis Competitive	mx	IG = 11 CG = 11	13.8 ± 1.7 for entire sample	Not assessed	Y-shaped Human No	12 weeks BATAK Pro™ training, twice a week	The BATAK Pro™ training program did not elicit statistically significant changes in agility performance.
(56) Journal article	Soccer Second division	m	IG = 24	17.8 ± 0.7	Not assessed	SpeedCourt Generic No	7 weeks SpeedCourt training, once a week	Significant differences between pre and post measurements were observed in AT left and AT right (*p < * 0.001, η^2^ = 0.54, *d* = 0.86; *p* <0.001, η^2^ = 0.73, *d* = 1.22, respectively).
(57) Journal article	Soccer Second division	m	*n* = 19 IG = ? CG = ?	14 ± 0.6 for entire sample	Not assessed	SpeedCourt Generic No	3 weeks repeated multi-directional sprint training (IG) or repeated shuttle sprints (CG), twice a week	IG improved agility performance compared to pre-test values (*p* <0.01, *g* = 1.03) and compared to CG (*p* = 0.01, *g* = 1.29).
(58) Journal article	Soccer First division	m	IG1 = 11 IG2 = 11 CG = 10	14.5 ± 0.9 for entire sample	Not assessed	Y-shape, with and without ball dribbling Human No	Agility (IG1) or CODS (IG2) training for 6 weeks, twice a week	A significant group effect was observed for AT with and without the ball (*F* = 10.35, *p* <0.01, η^2^ = large; *F* = 15.86, *p* <0.01, η^2^ = large, respectively). Group differences in post-test values of AT with (*p* <0.05) and without ball (*p* <0.05) were evident in IG1. Improvements of AT with and without ball were higher in IG1 than in IG2 (−7.73 ± 2.66%, *d* = 2.99; −9.37 ± 3.93%, *d* = 2.28 vs. −5.00 ± 1.26%, *d* = 1.03; −4.59 ± 3.43%, *d* = 1.09, respectively).
(53) Journal article	Soccer First division	m	IG1 = 12 IG2 = 12 CG = 12	14.2 ± 0.9 for entire sample	?	Y-shape, with and without ball dribbling Human No	SSG (IG1) or CODS (IG2) training for 6 weeks, 3 times per week	Significant main effects for time and groups were evident for AT with and without ball. Significantly higher improvements in AT with ball were found in IG1 compared with IG2 (*p* ≤ 0.05) and CG (*p* <0.01).
(62) Journal article	Soccer Highly trained	m	IG1 = 18 IG2 = 16	13.2 ± 1.2 13.4 ± 0.8	Not assessed	Y-shape Generic No	6 weeks flywheel eccentric overload training (IG1) or reactive strength training (IG2), twice a week	Significant differences over time and an interaction effect were observed in AT [*F*_(1, 40)_ = 42.88, *p* <0.001, η^2^ = 0.52; *F*_(1, 40)_ = 8.0, *p* <0.01, η^2^ = 0.17, respectively].
**Study Publication Type**	**Sport level**	**Sex**	**Group** ***n***	**Age (years)**	**Age from PHV (years)**	**Agility test Stimuli AT**^a^ **and RT**^b^ **separated**	**Treatment**	**Main findings**
(63) Journal article	Rugby union ?	m	IG1 = 10 IG2 = 10 CG = 10	14.6 ± 1.09 for entire sample	Not assessed	1 vs. 1 agility test Human Yes	Watching training videos with implicit (IG1) or explicit (IG2) information, single training session	A significant time effect was observed in RT [*F*_(1, 25)_ = 7.40, *p* =0.012]. No interaction effect was evident. *Post-hoc* comparisons revealed significant differences in RT from pre to post in IG1 and IG2 [*F*_(1, 25)_ = 5.27, *p* =0.030; *F*_(1, 25)_ = 4.30, *p* = 0.049, respectively] but not in CG (*p* > 0.05).
(74) Journal article	Badminton ?	mx	IG = 10	Junior high school	Not assessed	Visual reaction system for badminton Generic Yes	3 weeks of footwork drills, 3 times per week	Visual reaction time but not AT significantly improved after 9 sessions of footwork drills (*t* = 4.09, *p* <0.05; *t* = 1.71, *p* > 0.05, respectively).
(55) Journal article	Soccer Highest youth division	m	IG = 18 CG = 16	14.4 ± 0.4 14.4 ± 0.5	Not assessed	Y-shaped Human No	6 weeks of video-based training, twice per week	A significant main effect of time and a time × group interaction for AT was observed [*F*_(1, 32)_ = 12.1, *p* <0.001; *F*_(1, 32)_ = 4.4, *p* <0.05, respectively]. IG improved significantly in AT (*p* = 0.001) but not in the CG (*p* > 0.05).
(50) Journal article	Soccer Second division	f	IG1 = 9 IG2 = 10	19.0 ± 0.5 19.6 ± 0.5	Not assessed	Modified 20-m shuttle run Video No	5 weeks of resistance training on an unstable (IG1) or stable surface (IG2), 3 times per week	A significant main effect for time was found for AT (*p* <0.001). Both groups performed post-training significantly faster in the agility test (*p* <0.001).
(49) Journal article	Rugby league football High development level	m	IG = 8 CG = 7	18–19 for entire sample	Not assessed	Y-shape Video Yes	3 weeks of agility drills with concurrent video training, twice per week	Agility performance and perception and response time was significantly improved in the IG (*p* <0.05) but not in the CG.
(60) Journal article	Soccer Sub-elite	?	IG = 20 CG = 15	10.5 ± 0.3 10.7 ± 0.2	−2.58 ± 0.2 −2.48 ± 0.1	Y-shape Human No	SAQ training for 12 weeks, twice per week	An interaction effect was evident in AT [*F*_(1, 33)_ = 4.74, *p* <0.05, η^2^ = 0.12]. Improvements in agility performance were higher in IG compared to the CG (ES = 0.8, ES = 0.2, respectively).
(54) Journal article	Australian rules football Highest junior competition	m	IG1 = 13 IG2 = 12	17.5 ± 0.8 17.3 ± 0.5	Not assessed	Y-shape Video Yes	11 sessions of SSG (IG1) or CODS (IG2) training within a 7-week period	Group × time interactions were observed for AT (*p* <0.05) and RT (*p* <0.01). AT and RT were significantly improved after the training period in IG1 but not in IG2 (*p* <0.01, *d* = 0.93; *p* <0.001, *d* = 2.32; *p* > 0.05, *d* = 0; *p* > 0.05, *d* = 0.16, respectively).
**Study Publication Type**	**Sport level**	**Sex**	**Group** ***n***	**Age (years)**	**Age from PHV (years)**	**Agility test Stimuli AT**^a^ **and RT**^b^ **separated**	**Treatment**	**Main findings**
(70) Journal article	Soccer Elite	m	IG = 10 CG = 10	17.7 ± 0.4 16.8 ± 0.7	Not assessed	180° turn agility test Generic Yes	6 weeks of neuromuscular training, twice per week	A significant group × time interaction was evident for agility movement time (*p* <0.05, *d* = 0.33). *Post hoc* tests revealed significant improvements in IG (*p* <0.05, *d* = 0.97) but not in CG (*p* > 0.05).

AT, agility time; CG, control group; CODS, change-of-direction speed; ES, effect size; f, female; IG, intervention group; m, male; mx, mixed group of both sexes; PHV, peak height velocity; RT, response time; SAQ, speed, agility, and quickness; SSG, small-sided games; ?, information not provided.

a Time to fully complete the agility test.

b Timeframe from the onset of the stimulus until the initiation of the response movement.

Soccer (50–53, 55–58, 60–62, 65, 66, 68, 70–73, 77, 79–84) was the most examined sport, with over two-thirds (*n* = 25) of the 37 included studies approaching it. Athletes of other sports, such as futsal (76), Australian rules football (54), rugby league football (49), rugby union (63), badminton (74), and table tennis (59), were assessed in single studies each. Three studies included athletes of different sports (64, 75, 79), one included inactive subjects (69), and two studies did not report subjects' sport participation (32, 67).

### Subjects' characteristics

A total of 3,087 subjects were involved across the studies, with a sample size range of 20–553 subjects (*Mdn* = 72) for observational studies and 10–36 subjects (*M* = 25) for experimental studies. The majority (62.2%, *n* = 23) of studies included subjects of solely male participants (49, 52–58, 62–65, 68–70, 72, 73, 76–78, 80, 81, 83), whereas 5.4% (*n* = 2) included solely female subjects (50, 59), 13.5% (*n* = 5) included mixed groups of both sexes (32, 67, 74, 75, 79)), and in 18.9% (*n* = 7) subjects' sex was not reported at all (51, 60, 61, 66, 71, 82, 84). Only one study (32) comprised subject groups below 10 years of age. Maturation was estimated in eight cases (22%) (52, 53, 60, 71–73, 77, 84), applying methods of Tanner (86) (*n* = 1) and non-invasive estimates of Mirwald et al. (87) (*n* = 4) and Moore et al. (88) (*n* = 3).

### Agility assessments' characteristics

A total of 39 agility tests were employed, with presentations of generic (*n* = 27) (32, 51, 52, 56, 57, 59, 61, 62, 64–77, 80–84), video (*n* = 4) (49, 50, 54, 83) and human stimuli (*n* = 8) (53, 55, 58, 60, 63, 78, 79, 81). All agility tests that used sport-specific stimuli were designed to simulate defensive scenarios. A few studies (53, 58, 83) also included attacking scenarios due to subjects' ball possession, including trials with ball dribbles. However, these studies did not clearly state if the ball carrier's movement direction was opposed to the direction of the stimuli. Response times (timeframe from the onset of the stimulus until the initiation of the response movement) were separately assessed in 17.9% (*n* = 7) of cases (49, 54, 61, 63, 70, 74, 78). The Y-shaped agility test and its modified variants were the most frequently applied tests (*n* = 18) (49, 52–55, 58, 60–62, 67, 71, 73, 76–79, 81, 84). Agility tests differed regarding cutting angles, distances covered, number of consecutive CODs, number of COD options, starting velocities, dribbles, and other characteristics.

### Outcomes of observational studies

In most studies, the youngest group (range: 7-16 years) of athletes was outperformed in agility by the group with the oldest age (range: 14–18 years) (51, 52, 61, 65, 68, 69, 71–73, 78, 82–84). A few other studies did not find differences in agility performance among age groups (59, 76, 81). Differences were rather evident between younger age groups than between groups of older athletes (61, 69, 72, 82–84).

Maturation was identified as a factor affecting agility performance across ages as observed age group differences were no longer evident when maturation was included as a covariate (52, 72). In addition, significant correlations of trivial to large magnitudes between agility and measures of CODS, sprint, and jump performance were reported (51, 52, 61, 66, 68, 72, 73, 77, 80, 84). Studies calculating regression analyses revealed several predictors of agility performance, such as maturation (adjusted *R*^2^ = 8%) (52), sex (β = −0.46), age (β = −0.30), training experience (β = −0.18) (75), and in-line lunge performance (adjusted *R*^2^ = 38%) (52).

### Outcomes of experimental studies

The 14 experimental studies comprised 21 intervention groups that were exposed to 15 different training programs. Table 3 gives an overview of the various training programs and their elicited effects in the respective intervention groups. Training programs can be categorized regarding their specificity to agility: Training involving neither unplanned movements nor any perceptual-cognitive elements is considered training programs of low specificity (e.g., COD drills, resistance training) (50, 53, 54, 57, 58, 62). Training programs exhibit a medium specificity if either unplanned movements or perceptual-cognitive elements are involved (e.g., video-based perception training) (55–57, 63, 74, 79). In contrast, a high specificity is achieved if both are included (e.g., agility drills, small-sided games) (49, 53, 54, 58, 60, 70). Most intervention groups experienced significant improvements in agility performance to different extents (49, 50, 53–58, 60, 62, 63, 70). Nevertheless, two groups of the moderate specificity category (74, 79) and three groups of low specific training programs (54, 57, 62) failed to improve agility performance.

**Table 3:** Overview of training programs and respective outcomes.

***n***	**Training program**	**Study**	**Specificity level**	**Pre-post changes (%)**	**Change in agility performance (ES, 95% CI)**	**Age (years)**	**Control group**	**Comments**
1	Agility drills	(58)	High^a^	−9.4	↑ 2.28 [1.21, 3.35]^*^	14.5 ± 0.9	Yes	CG significantly improved
1	Agility and video training	(49)		−5.8^*^	↑ 0.69* [−0.32, 1.70]^*^	18–19	Yes	RT significantly improved
1	Neuromuscular training	(70)		−4.7^*^	↑ 0.85* [−0.07, 1.77]^*^	17.7 ± 0.4	Yes	Only generic test stimuli, no significant improvement in RT
1	SAQ training	(60)		−4.2	↑ 0.8 [0.16, 1.44]^*^	10.5 ± 0.3	Yes	
2	Small-sided games	(53)		−4.8^*^	↑ 1.38* [0.49, 2.27]^*^	14.2 ± 0.9	Yes	CG significantly improved
		(54)		−3.8	↑ 0.93 [0.12, 1.74]^*^	17.5 ± 0.8	No	RT significantly improved
1	BATAK Pro™ training	(79)	Moderate^b^	2.5	→	13.8 ± 1.7	Yes	
1	Footwork drills	(74)		−14.4^*^	→	JHS	No	RT significantly improved
2	SpeedCourt training	(56)		−5.9 to −10.5^*^	↑ 0.86 to 1.22 [0.27, 1.84]^*^	17.8 ± 0.7	No	
		(57)		−9.9	↑ 1.03 [0.07, 1.99]^*^	14.0 ± 0.6	Yes	
3	Video-based training	(55)		−12.6	↑ 0.85* [0.17, 1.58]^*^	14.4 ± 0.4	Yes	
	(Explicit cues)	(63)		−19.1	↑ 0.28 [−0.60, 1.16]^*^	14.6 ± 1.1	Yes	Outcome measure is RT
	(Implicit cues)	(63)		−15.7	↑ 0.33 [−0.55, 1.21]^*^	14.6 ± 1.1	Yes	Outcome measure is RT
3	COD drills	(58)	Low^c^	−4.6	↑ 1.09 [0.19, 1.99]^*^	14.5 ± 0.9	Yes	CG significantly improved
		(53)		−3.6^*^	↑ 0.57* [−0.25, 1.39]^*^	14.2 ± 0.9	Yes	CG significantly improved
		(54)		0	→	17.3 ± 0.5	No	
1	Flywheel eccentric training	(62)		−9.1^*^	↑ 1.86* [1.08, 2.64]^*^	13.2 ± 1.2	No	
1	Reactive strength training	(62)		−2.9^*^	→	13.4 ± 0.8	No	
1	Repeated shuttle sprints	(57)		−2.2	→	14.0 ± 0.6	No	This group served as CG
1	Resistance training	(50)		−6.6^*^	↑ 0.96* [0.03, 1.89]^*^	19.6 ± 0.5	No	
1	Unstable resistance training	(50)		−7.7^*^	↑ 1.00* [0.02, 1.98]^*^	19.0 ± 0.5	No	

CG, control group; COD, change-of-direction; ES, effect size; JHS, junior high school; RT, response time; SAQ, speed, agility, and quickness; ↑, significant improvement; → , no significant change.

a Training involves unplanned movements and anticipative elements.

b Training involves either unplanned movements or anticipative elements.

c Training involves neither unplanned movements nor anticipative elements.

* *Post-hoc* calculated by the authors.

## Discussion

This systematic scoping review aimed to (1) to map the literature approaching agility in the youth population, (2) to identify research gaps, and (3) to outline the existing literature regarding the trainability, “natural” development, and contribution of underlying key factors of agility performance in consideration of maturation. A total of 41 reports that explored agility in youth were systematically identified. Outcomes are discussed in detail, and recommendations for practice and research are made in the following subsections.

### Current pediatric agility literature

The rising number of publications per year indicates an increasing interest in the present topic. However, the completed data acquisition might explain the low number of publications in 2021 by midyear and by the COVID-19 pandemic, which potentially reduced research activity. Surprisingly, despite the early contemplation of agility as a movement in response to perceived stimuli in 1976 by (89), the first article examining this agility concept in youth was published in 2011 (49), indicating a fairly young body of literature. Although 41 reports approaching agility in youth were identified, the extent of evidence is still sparse, especially if specific research questions are sought to be answered.

Biases in the literature were detected regarding countries of origin (i.e., mainly European countries) and subjects' age, sex, and practiced sport (i.e., above 10 years of age, mostly males, and soccer, respectively); this limited the range of evidence. In addition, the lack of research on female subjects is problematic since results obtained in boys might not unconditionally be transferred to girls due to sex and maturation interaction in components of motor performance (90). Furthermore, most studies assessed agility in over 10-year-olds, which leaves questions concerning agility training in childhood and its meaningfulness regarding long-term athletic development unanswered (7, 18). Consequently, more extensive and diverse research is warranted to address these deficiencies to expand the range and extent of evidence.

The nature of the evidence is characterized by observational (cross-sectional) and experimental studies and secondary research. Included secondary research of (5), (39), and (30) examined the “natural” development and trainability of agility in young athletes. However, due to the lack of literature, assumptions of “natural” development of agility and given training recommendations were predominantly based on inferences deduced from CODS research and research of the particular determining factors of agility performance. Secondary research of (85) dedicated a section of their monograph to the “natural” development of agility. However, its content is almost entirely based on an also included study of the same authors (32).

Meta-analyses of training effects seem relatively meaningless considering the wide range of methods of included studies. Especially, the applied agility tests differ regarding stimulus presentation, cutting angle, distance covered, direction change from a stopping position or out of the movement, and the number of options and direction changes. This complicates direct comparisons of study results applying different tests since agility is considered a context-specific entity, thus dependent on task constraints (27).

Although a quality assessment is not provided in this scoping review, existing methodological and research design limitations of included literature should be acknowledged, such as the relatively small sample sizes in the experimental studies with a mean sample size of *n* = 13 subjects in the intervention groups, the absence of control groups in 5 of 14 studies, and the scarcely assessed response time limiting interpretation of training effects. Moreover, maturation was rarely estimated, making it difficult to draw theoretical conclusions about maturation effects.

The included studies assessed agility in defensive scenarios. However, evidence suggests that attacking and defending agility are distinct skills (91, 92). In invasion sports, agility movements in attackers vs. defenders clearly differ in regard to their goals (e.g., chasing or evading), strategies (e.g., execution of deceptive actions), imposed physical demands, performed COD techniques, and perceptual information used (15, 91–94). Thus, it should be noted that the presented results might not apply to offensive agility.

### Determining factors of agility performance

Current agility models identified various performance-determining factors such as perceptual and decision-making factors, technical qualities, and physical features (4, 9, 13, 24–27). However, the contribution of these factors depends on the imposed agility task and on the environment in which it is performed (95). Moreover, studies suggest that the relationships between agility performance and physical qualities are dependent on athletes' age.

Moderate to high relationships between jump and agility performance and high relationships between sprint and agility performance was observed in U11–U14 age groups (51, 84). High correlations between CODS and agility performance were found in U11–U13 age groups (66, 72, 84). The rather high correlations observed might result from the applied generic stimuli in the agility tests. Generic stimuli prohibit the usage of some perceptual and cognitive factors (e.g., pattern recognition, anticipation). Thus, a greater demand is placed on physical abilities to execute the agility tasks (84). The influence of physical capacities is even more pronounced in CODS, in which perceptual and cognitive demands are mostly omitted. Therefore, physical qualities are generally more associated with CODS than agility performance (10, 11, 77, 96–99).

However, as correlation coefficients tend to decline or parameters are not significantly correlated at all, several authors reported decreasing associations between physical capacities (i.e., jump, sprint, and CODS performance) and agility performance with escalating age groups (51, 61, 66, 72, 83, 84). This is not surprising since, in adulthood, physical qualities and agility are less related (97–101), and CODS and agility are even considered distinct skills (10–17) and thus, approaching a fully mature state ceases their relationship. The notion that factors other than physical capacities (i.e., technical, perceptual, and cognitive factors) are increasingly related to agility performance in older age groups is supported by findings of a stagnant sprint, jump, and CODS performance concomitant with enhanced agility performance in U17–U19 athletes (68, 78). Movement technique, perceptual, and decision-making factors have a higher relevance for agility performance in adults (100). Conversely, in young athletes, physical qualities seem to be more determinant. Due to their age, younger athletes probably exhibit less game experience and shorter training history, resulting in lower perceptual and decision-making skills, inexperienced technique, and lack of movement strategies than older athletes (51, 61, 102–104). This is also reflected by higher response times in younger athletes (61).

Nonetheless, (73) stated that the relationships between agility and sprint, jump, and CODS performance were more pronounced in U15 than in U13 athletes, conflicting with the above-mentioned findings. The authors supposed that the older group possesses a higher level of technical skill due to longer involvement in training, which enables them to exploit their sprinting and jumping qualities to a greater extent and convert them into effective agility maneuvers.

Further impacting factors apart from the discussed organismic factors were observed. For example, age and gender, training frequency, training experience, body mass in girls, and peripheral perception in boys were identified as contributing or impairing factors (75). In addition, several measures of movement proficiency of the functional movement screen (in-line lunge, deep overhead squat, active straight leg raise, and rotary stability) were found to be related to agility performance (52).

### “Natural” development of agility

In most studies, higher agility performances in ascending age groups were observable, indicating a “natural” development of agility (51, 52, 61, 65, 68, 69, 71–73, 78, 82–84). However, significant differences were found between groups with higher age differences than between adjacent age groups. The enhancement of agility performance through the years is confirmed by regression analyses of (75), who observed a significant influence of chronological age on agility test performance.

The included observational studies are exclusively conceptualized in cross-sectional designs, accompanied by several limitations. First, cross-sectional designs do not capture individual developmental trajectories as subjects are generally assessed only once. Furthermore, age groups are arranged regarding the chronological age of the subjects. This might disguise differences between age brackets due to a potentially high intersubject variability within the age groups caused by different biological ages (i.e., maturation stage) of the peers, especially in groups with a broad age range (105). Hence, the less pronounced performance differences between adjacent age groups might be explained because maturation was rarely controlled. Therefore, athletes of different biological ages were pooled in the same chronological age group.

(52) and (72) showed that age group differences disappeared when maturation was entered as a covariate, emphasizing that maturation is one of the main contributing factors for differences between age groups. Although, contrary results were observed by (71). In this study, age group differences remained evident after adjusting to maturation, suggesting that not solely maturation but also different training regimes across age groups are probably responsible for differences in agility performance. In addition, data from a recent study described a merely moderate relationship (*r* = −0.36) between maturation and agility performance (77).

Longitudinal data of male soccer players indicates that CODS naturally improves with increasing age with a non-linear trajectory culminating between 13 and 14 years of age and a leveling off at the age of 17 (106). Similar results were found for agility performance in a cross-sectional study of (32) conducted on girls and boys between 7 and 18 years, albeit with a divergent slope. The increments in agility performance were rather high across childhood, but the increases diminish with ascending age groups and almost plateau in the group of 14-years-olds. The absence of a pronounced peak and the early flattening of the curve compared to the trajectory of CODS development might be explained by the mixed sample of girls and boys, who mature with different timings and thus, smoothen the curve due to higher inter-subject variability within the age groups. Unfortunately, this study is the only source providing insights into agility performance in children below 10 years of age. Longitudinal data on CODS in children suggests that changes in CODS performance depend on the mid-childhood growth spurt, which varies interindividually in terms of timing and tempo (21), thus, indicating a non-linear development of CODS (107). If this also applies to agility development in childhood is yet to be explored.

The above-mentioned stagnant agility performance starting at the age of 14 aligns with (82), who did not observe differences in agility performance in U15, U16, and U17 soccer players. Comparable results were obtained in a study of male soccer players who demonstrated differences in agility performance between age groups ranging between U12 and U16. In contrast, no changes were evident between U16 and U18 players (72). Stagnant periods were also observed in other studies (52, 59, 61, 69, 78, 81, 83, 84), albeit at different ages at entry. As opposed to this, (71) and (65) could not corroborate these findings as all age groups (U12-U16 and U10-16, respectively) differed significantly regarding agility performance in their observations. An explanation of stagnant periods in performance, although speculative, provides the concept of “adolescent awkwardness”, which refers to a temporary decline in performance or a disruption of motor coordination in adolescents as a consequence of rapid growth (20, 105, 108). This phenomenon typically emerges ~6 months before the zenith of the adolescent growth spurt (also known as peak height velocity [PHV]), which occurs at about 12 years in girls and 14 years in boys (109, 110). However, not all children experience delays or regression in performance (105).

Conflicting results were reported concerning the development of agility in late adolescence. Studies in male soccer players did not observe differences in agility performance between U19 and U17 (81) and U15 (83) age groups. In addition, adult (mean age: 28.1 ± 5.2 years) futsal players did not show higher agility performance than U19 players (76). Whereas, in studies of (68) and (78), U19 players outperformed U17 male soccer players in agility performance. Since no differences in sprinting speed, reactive strength, or jumping performance were observed between these age groups, the authors concluded that differences in agility performance were rather attributable to superior perceptual, anticipatory (78), and technical skills (68) than to enhanced physical capacities. Thus, differences were presumably predominantly induced by training and gained expertise and less by consequences of maturation (72). It might be assumed that growth- and maturation-induced differences in agility performance between age groups recede in late adolescents until they completely cease when achieving a fully mature adult state (76).

### Trainability of agility

Due to the multidimensional nature of agility, there is a broad spectrum of training methods to enhance this skill (111). Although movement specificity and the representative design of practice are related to transfer, training can be remote to the target movement and sporting context and still be capable of improving agility performance (112). Thus, it is not a strict necessity to train agility as a whole, and improvements can also be attained by training individual components of agility (113).

The 14 included experimental studies evaluated the effects of various training interventions on agility performance. Significant improvements in agility performance were observed in 16 of 21 training approaches. The chosen training approaches differed regarding their specificity to agility movements and were classified into training programs of high, moderate, and low specificity (Table 3). Training programs of high specificity aim to train agility skills as a whole. In contrast, training interventions in the category of moderate specificity are characterized by the involvement of either unplanned movements or specific cognitive components. Training programs of low specificity neither involve unplanned movements nor perceptual or decision-making elements.

#### Training programs of high specificity

Agility movements are performed in response to a stimulus, whereby stimulus perception and execution of the response movement are interdependent and constitute a coherent entity (i.e., perception-action coupling). It was argued that a decoupling of perception and action might impair the transfer of training/learning to the whole agility skill (14, 114). Therefore, to maintain the integrity of the agility skill, representative tasks used in perceptual-cognitive skills training should replicate real-life performance as closely as possible (114). Likewise, sport-specific decision-making elements should be included in physical training if the movement component of agility is sought to improve (14).

In a video-based training by (49), subjects had to change movement direction in response to video footage of an attacking opponent, whereby perception-action coupling was maintained. Agility performance significantly improved, which was attributed to enhanced perception since response time improved while performance in the pre-planned test condition remained the same. Supportive findings were obtained in a study of junior soccer players in which video-based tactical training combined with sport-specific motor responses led to improved response times, response accuracy, and ball kicking movement times in a tactical decision-making test (115).

In three interventions, exercises with persistent perception-action coupling (e.g., catching/evading duels, mirror drills, ball pass in a random direction) were utilized (58, 60, 70). An intervention group practicing this training form increased linear sprinting, CODS, and agility performance in the study of (58), whereby gains in agility performance were significantly higher in comparison to gains of a CODS training group. However, results should be interpreted carefully since improvements in performance parameters were also evident in the control group. (60) and (70) agreed with findings of enhanced agility performance showing large effect sizes. In the latter study, applying a generic stimulus in the agility test possibly concealed potential improvements in anticipation performance since no changes in response time were observed.

Another way of retaining perception-action coupling and a high representativity of the training task is exercising in small-sided games in which task constraints such as field dimensions, number of players, and rules are modified (116). (53) showed an enhanced agility performance accompanied by increases in sprint, jump, and CODS performance after a training intervention of small-sided games. Gains in agility performance were superior in the intervention group practicing small-sided games compared to the COD-exercising group. Nevertheless, performance gains were partly attributed to concomitantly conducted soccer training since increases were also observed in the control group. In the study of (54), small-sided game training in U18 Australian Rules footballers significantly improved agility performance, eliciting a large effect. Improvements were exclusively due to enhanced perception and decision-making since response time was reduced, whereas movement response time remained unchanged.

#### Training programs of moderate specificity

Subjects in the training interventions of (56), (57), and (74) had to perform unplanned multi-directional movements in response to random generic visual cues indicating movement direction. Improvements in agility performance were solely evident in the studies of (56) and (57), displaying large effects. In the latter study, measures of physical qualities (i.e., vertical jump and sprint) did not improve, suggesting that gains in agility performance are possibly due to specific technical enhancements. Randomly lit-up LED targets had to be hit in the BATAK Pro™ training in the study of (79). An improvement in agility performance was not evident, probably due to a lack of specificity of the training movements and the generic light stimulus with the demands of the utilized agility test.

Research suggests that perception and decision-making skills are trainable in youth (31). Several studies have proven that video-based training can enhance these skills in children and adolescents (115, 117–119). For example, (119) observed improved decision accuracy and faster decision times after video-based perceptual training in young softball players, which also transferred to the field environment. These findings align with the included study of (55), in which faster and more accurate decisions induced by video-based training positively transferred to agility performance. Furthermore, (63) employed video-based warm-up interventions with either an explicit (highlighted key kinematic cues) or an implicit (no additional information given) learning strategy which both resulted in faster response times in an agility test. Likewise, prepubescent tennis players could improve their anticipation skills irrespective of the applied learning strategy (118).

#### Training programs of low specificity

The category of low specificity comprises CODS, reactive strength, and resistance training approaches that predominantly target enhancing physical capacities to improve agility performance eventually. Physical qualities and agility are less related in adults (97–101), whereby the potential of transfer of corresponding training programs to agility performance might be reduced. Associations between physical factors and agility performance are generally stronger in youth athletes, especially in younger juveniles. Thus, the positive transfer of training approaches targeting physical capacities is probably seen in younger athletes. A reasonable amount of evidence suggests that resistance training can considerably increase strength in children and adolescents (28, 29, 120). Furthermore, a recent umbrella review proved that resistance training-induced gains in strength have the potential to positively transfer to CODS performance beyond a level achievable from growth and maturation alone (121). In line with this, (50) showed large effects of regular and unstable resistance training on agility performance in female soccer players. In addition, (62) observed large effects of eccentric training with a flywheel device on agility performance. Isoinertial eccentric training has previously shown the potential to enhance CODS in young soccer athletes, according to recent findings (122, 123). Research suggests that reactive strength training can improve COD ability in youth (124), albeit with more significant gains in older youths (125). This was not observed for agility performance in the study of (62) since a reactive strength training intervention did not elicit significant changes in agility performance.

In contrast to the mentioned strength training forms, the directional change is actually performed in COD drills. Even though the movement is executed pre-planned, it resembles the COD movement performed in unplanned agility tasks and thus, is potentially more specific than resistance training exercises. Nevertheless, the included training interventions employing COD drills and repeated shuttle sprints show ambiguous results. Improved CODS performance after COD training interventions were evident in three of four studies (53, 57, 58). However, only in studies by (53) and (58) did gains in CODS transfer positively to agility performance. It must be noted that in both studies, the control group also experienced significant improvements. In the study of (54), the training group performing COD drills did not improve in CODS or agility performance.

In conclusion, the results of positive transfer of different training forms to gains in agility performance lead to the assumption that agility is indeed trainable in youth, even though the underlying mechanisms are disparate. Nevertheless, the heterogeneity of applied training procedures, performance tests, training goals, and subjects' ages, performance levels, and exploitation of performance limits reduce the comparability of study outcomes and complicate deeper analyses of how maturation affects trainability of agility and ultimately, how agility should be trained at a particular age.

### Limitations

This scoping review has limitations that need to be addressed. First, only reports written in English or German were considered due to feasibility reasons, which potentially led to disregarded knowledge published in other languages. Moreover, following the recommendations of (44), included studies were not critically appraised. Notwithstanding, researchers are advised to appraise the quality of these studies if a more specific research question is about to be answered.

## Future directions and recommendations

Considering the importance of agility for sports performance, the unabated desideratum of agility research in the youth population, and the advancing research activity in this field, agility research in youth has undoubtedly the potential to become a Cinderella story in pediatric exercise science, rising from neglect to being a hot topic. Nevertheless, the extent and range of the current body of literature are still limited, indicating an abundance of research gaps that are yet to be closed. Further agility research could provide a deeper understanding of the trainability of agility by unraveling the involvement of underlying key factors with due regard to biological age. This could help practitioners design evidence-based strategies for agility development throughout childhood and adolescence, which constitute an integral component of an all-embracing long-term athletic development (7, 39).

In this light, several recommendations can be made to guide future research endeavors:
More diverse research is postulated to expand the range of evidence, i.e., research in middle childhood, female subjects, and sports other than soccer.
Research designs with cross-sectional chronological age group comparisons have inherent deficiencies (105). Longitudinal study designs can offset these limitations and are endorsed when examining developmental effects.
Considering the influence of maturation on agility performance, it is advised to employ appropriate methods to estimate and control the maturation stage in youths.
Generic stimuli in agility tests disregard perceptual-cognitive factors of agility performance. This should be considered especially when testing older youths since perceptual-cognitive factors are increasingly determinant to agility performance with progressing age. Aside from the fact that representative design of agility practice and testing are endorsed in general (14, 36, 38, 126, 127), sport-specific stimuli should be employed in particular if agility tests are utilized to assess the effects of training approaches targeting the improvement of perceptual-cognitive factors.
Adults show distinctive perceptual-cognitive and biomechanical differences between attacking and defending agility (91–93). These differences are likely to be seen in young athletes as well, which needs further investigation. Thus, training and testing of athletes should be specifically designed for both conditions (15, 92–94, 128).
Outcome measures of agility performance in the included studies were exclusively time-based. Since not all in-game agility events happen at maximal speed (15, 113), a qualitative, more direct, and possibly more task-representative approach is to assess the immediate outcome of an agility event (e.g., successful or failed evasion or tackle) (92). In addition, athletes could potentially better exploit their repertoire of agility actions in outcome-based tests (e.g., successive application of different agility techniques or feints). Further research is required to assess the feasibility of such outcome-based agility testing in young athletes. Thus, practitioners and researchers should consider carefully whether an outcome-related or time-based measure best represents agility performance success (129).
Comprehensive assessments of determining factors are necessary to draw inferences about the origins of changes in agility performance (38). Those assessments are especially relevant for practitioners to expose athletes' strengths and deficits for adequate training prescription (96). Although physical performance parameters were frequently measured in conjunction with agility assessments, perceptual-cognitive factors (e.g., estimated by response time) were less often assessed. Surprisingly, technical qualities were not assessed, probably due to the absence of validated screening tools to assess COD technique regarding performance (26). Nonetheless, technical qualities in terms of appropriate sequencing of muscle actions, adoption of an appropriate body position, and coordination of force and impulse are requirements for fast agility movements (15, 27). Therefore, qualitative analyses of the COD technique can provide highly valuable information for the practitioner (129). Furthermore, since motor coordination explains a significant amount of variance in agility performance in adolescents (130), it might be assumed that technical qualities are similarly important for agility performance.

## Conclusion

A decade ago, agility was described as one of the most under-researched fitness components within the pediatric literature (7). Today, agility research in youth might still be in its infancy, but the progressively accumulating evidence base over the years evinces an increasing research activity and interest in this topic.

This systematic scoping review is the first mapping of the body of literature about agility in youth to the best of our knowledge. Its research questions aimed to examine and synthesize the current state of knowledge about trainability and “natural” development of agility. Present key findings may have implications for researchers and practitioners and can be outlined as follows: Evidence suggests that agility performance is influenced by chronological and biological age and thus, “naturally” improves throughout childhood and adolescence in a non-linear manner. Furthermore, various training approaches with different underlying mechanisms could enhance agility performance confirming the trainability of agility in youths in general, even though influences of maturation remain cloudy. Moreover, results indicate that with progressing age, perceptual-cognitive factors are increasingly associated with agility performance, whereas relations with physical factors diminish. The relationship between technical factors and agility performance remains unknown and needs further investigation.

In the light of the significance of agility for sports performance, future research in this field is postulated to provide practitioners with evidence-based recommendations for maturation-dependent training prescription. This will help pave the way for an overarching long-term athletic development strategy, with agility as one of its integral components.

## Data availability statement

The datasets presented in this study can be found in online repositories. The names of the repository/repositories and accession number(s) can be found below: https://osf.io/8y9nk.

## Author contributions

LT and DB contributed to the study conception, study design, the pre-registration of the study protocol, data analysis, and interpretation. LT contributed to literature search, screening and selection as well as performed the data charting, and wrote the first draft of the manuscript. All authors iteratively revised the manuscript until mutual approval of the final version.

## Funding

The authors gratefully acknowledge the support of the Deutsche Forschungsgemeinschaft (DFG) and the Open Access Publication Fund of the Library and Information System (BIS; Carl von Ossietzky University Oldenburg, Germany) for covering article processing charges.

## Conflict of interest

The authors declare that the research was conducted in the absence of any commercial or financial relationships that could be construed as a potential conflict of interest.

## Publisher's note

All claims expressed in this article are solely those of the authors and do not necessarily represent those of their affiliated organizations, or those of the publisher, the editors and the reviewers. Any product that may be evaluated in this article, or claim that may be made by its manufacturer, is not guaranteed or endorsed by the publisher.

## Footnotes

^1^ https://osf.io/73cxs

^2^ https://osf.io/8y9nk
